# Exercise and nutritional prehabilitation in gastrointestinal cancer patients: prospective controlled trial assessing feasibility, safety and effects on quality of life

**DOI:** 10.1186/s12885-026-16435-y

**Published:** 2026-06-30

**Authors:** Laurie Assenbaum, Antonia Pahl, Freerk T. Baumann, Cécile Weiß, Hartmut Bertz, Gabriel Seifert, Hannes Neeff, Christine Greil

**Affiliations:** 1 Department of Medicine I, Medical Center-University of Freiburg, Faculty of Medicine, University of Freiburg, Freiburg, Germany; 2 Department I of Internal Medicine, Working Group Oncological Exercise Medicine, University Hospital Cologne, Center for Integrated Oncology Aachen Bonn Cologne Düsseldorf, Cologne, Germany; 3 Department of Visceral and General Surgery, University Medical Center Freiburg, Freiburg, Germany; 4 Department of Visceral and General Surgery, Diakonie Klinikum Stuttgart, Stuttgart, Germany

**Keywords:** Gastrointestinal neoplasms, Preoperative exercise, Nutrition therapy, Feasibility, Quality of life

## Abstract

**Background:**

Perioperative morbidity in gastrointestinal (GI) cancers is closely associated with reduced physical fitness and impaired nutritional status. While prehabilitation has been shown to improve outcomes in patients with colorectal cancer (CRC), it is not yet standard of care and remains underexplored in other GI malignancies. This study evaluates the feasibility, safety, and preliminary effectiveness of a supervised moderate-to-high intensity exercise program combined with nutritional counseling in a cohort of GI cancer patients scheduled for surgery.

**Methods:**

In a prospective, two-arm, controlled trial, patients scheduled for GI cancer surgery were assigned to a prehabilitation program (2–3 sessions/week ≥ 3 weeks, endurance and resistance training with nutritional counseling) or usual care. Primary endpoints were feasibility (eligibility, recruitment, acceptance, retention, adherence) and safety (adverse events). Secondary endpoint was quality of life (QoL; EORTC QLQ-C30 global score, SF-36 physical and mental health component scores, PCS, MCS), assessed at baseline (t0), presurgery (t1), hospital discharge (t2), and 12-week follow-up (t3).

**Results:**

Among the 400 patients assessed for eligibility, 36% met the eligibility criteria. Of those approached, 41% consented to participate, resulting in an overall recruitment rate of 27% (*n* = 38). Of the recruited patients, 84% completed the study (*n* = 32; prehabilitation = 17; usual care = 15; mean age 63.5 years, range 38—85; ICD-10 C15-C26). Participants attended 95% of the planned sessions (8.1 ± 3.6) within a mean of 30 days (SD ± 16) and completed 59% at the target intensity. Nutritional counseling was provided to 94% of the patients. No intervention-related serious adverse events occurred. A modest improvement in PCS was observed in the prehabilitation group at t1 (+ 4.25 points), although this finding reached statistical significance only in the one-tailed analysis. No between-group differences were observed for global QoL or MCS.

**Conclusion:**

Multimodal prehabilitation combining supervised moderate-to-high intensity exercise with nutritional counseling is feasible and safe in a real-world GI cancer population. Recruitment and achievement of prescribed training intensity remain key challenges. Preliminary findings indicate short-term benefits for physical health, supporting further investigations in larger randomized trials.

**Trial registration:**

DRKS00028728; prospectively registered 05/05/2022.

## Background

Surgery remains a fundamental component of gastrointestinal (GI) tumor treatment. However, it is frequently accompanied by rapid declines in physical fitness, muscle mass, and nutritional status, which in turn increase postoperative morbidity and impair health-related quality of life (QoL) [1–3]. Enhanced Recovery After Surgery (ERAS) pathways reduce perioperative complications by standardizing analgesia, fluid management, and early mobilization, but they typically lack a structured exercise component beyond bedside ambulation [4].

Prehabilitation has emerged as a promising intervention for preoperative conditioning, particularly in the context of colorectal cancer (CRC) management [5]. The extent to which these benefits can be applied to other GI malignancies and the potential for integrating this approach into routine clinical care remain insufficiently clarified. The current evidence is characterized by limited clinical generalizability, largely due to a focus on single tumor entities. These factors undermine the representativeness of study samples and limit their transferability to real-world clinical settings [6]. Furthermore, prior work has been hampered by suboptimal reporting quality, limited consideration of patient-reported barriers, and intervention designs characterized by unimodal approaches, insufficient supervision, and training intensities unlikely to induce meaningful physiological adaptation [6]. Despite this, substantial heterogeneity persists regarding intervention type, intensity, and duration, precluding the formulation of precise criteria (FITT: frequency, intensity, time, type) for preoperative conditioning [7]. Furthermore, given the widespread availability of nutritional counseling, a multimodal strategy that integrates exercise and nutrition is particularly promising. The feasibility of delivering a moderate-to-high intensity program across the full spectrum of GI cancers, where surgical timelines are often short and symptom burden varies considerably, has not been systematically evaluated.

The present work was conceived as a preparatory feasibility study for a future definitive trial. The primary aim was to assess key feasibility metrics and the safety of a supervised, individualized moderate-to-high intensity exercise program combined with guideline-based nutritional counseling across a heterogeneous real-world GI cancer population. Secondary aim was to obtain data on patient-reported outcomes, including QoL, physical and mental health. Further exploratory analyzes included early survival outcomes, which may inform the design of future trials.

## Methods

### Study design and patients

We conducted a prospective, non-randomized, two-arm controlled feasibility study to evaluate the implementation of a structured prehabilitation pathway combining exercise and nutritional therapy in patients scheduled for GI cancer surgery. Data were collected by exercise scientists at the Department of Internal Medicine, University Medical Center Freiburg (Germany), between September 2022 and April 2024. Adult patients diagnosed with any type of GI cancer who were scheduled for surgery at least four weeks after enrollment were eligible for inclusion, allowing sufficient time to complete a minimum of three weeks of preoperative exercise training. Exclusion criteria comprised pregnancy, inability to undergo physical examination or participate in exercise training, participation in an organized strength-training program and relevant language barriers. Group allocation was based on patient preference and their ability to attend on-site training sessions multiple times per week. QoL was assessed at the time of surgical indication (t0), upon hospital admission (± 1 day, t1), at hospital discharge (± 1 day, t2), and at 12 weeks post-discharge (t3).

The analysis was pre-planned in two parts: The present paper focuses on feasibility outcomes of the intervention, including patient recruitment, adherence, program acceptance, safety, and effects on QoL in the overall cohort. Analyses of clinical outcomes, including functional recovery and nutritional status, were conducted separately and are to be reported in a separate manuscript which additionally focuses on CRC patients (Assenbaum et al., not yet published). The trial adhered to the guidelines of the Declaration of Helsinki and Good Clinical Practice (German Register of Clinical Trials No.: DRKS00028728). All participants provided written informed consent in line with the institutional review board guidelines (Ethics Committee Freiburg, Vote 22–1045).

### Intervention

The experimental intervention involved supervised, equipment-based aerobic and resistance exercises. The exercises were tailored individually for each patient and supervised by certified oncology exercise therapists in the hospital´s gym for oncology outpatients. The BORG scale was used to ensure moderate-to-high training intensity (BORG 13–17) [8]. Patients participated in this exercise program two to three times per week for one hour, with 15 min of aerobic exercise and 45 min of full-body resistance training, for a minimum of three weeks before surgery. Aerobic exercise was performed on a cycle ergometer, cross trainer or treadmill. The main strength machines used were “Leg press”, “Row”, “Chest” and “Back” (Cybex). In addition, patients received at least one session between 30 and 45 min of guideline-driven nutritional counseling prior to surgery [9], adapted to their nutritional status and individual energy and nutrient requirements. Nutritional counseling did not include nutritional supplementation. The control group received usual care, which included a general recommendation to remain physically active and access the hospital’s routine nutritional counseling services.

### Primary outcomes: feasibility and safety

The primary outcome feasibility was determined on the basis of quality criteria for feasibility exercise studies [6] and assessed by eligibility, recruitment, acceptance rate, retention, adherence, and adverse events. Eligibility rate was defined as the percentage of potentially eligible patients who were eligible for study consent. Recruitment rate was defined as the percentage of eligible patients who were recruited to participate. Acceptance rate was the proportion of approached patients who agreed to participate. Retention rates were evaluated in two ways: as the percentage of study completion either in relation to the recruited patients or in relation to the eligible patients. Adherence rate was defined as the percentage of completed exercise sessions in relation to the number of planned exercise sessions. The cutoff value for adherence was set at 70% according to previous literature [10]. Furthermore, the percentage of training sessions in the planned intensity was calculated. Program safety was measured by the number of adverse events caused by the intervention.

### Secondary outcomes: QoL (global QoL, PCS, MCS)

The secondary outcome QoL was assessed via two patient reported instruments. The EORTC QLQ C30 [11] is a standard instrument used in international clinical trials to assess QoL in oncology patients. It contains ten subscales, the functional scale global QoL is employed in this particular context. A high score on this scale ranging from 0 to 100 is indicative of a high QoL [11]. The SF-36 is a widely accepted instrument for the evaluation of subjective health that involves a physical and mental health component summary score (PCS and MCS) with higher scores indicating better outcomes.

### Further analyses: survival (OS, PFS)

Overall survival (OS) and progression-free survival (PFS) were assessed as exploratory outcomes and calculated from the date of cancer diagnosis including both initial and recurrent diagnoses. OS was defined as the time from diagnosis to death from any cause, PFS as the time from diagnosis to either documented disease progression or death, whichever occurred first. Patients without an event were censored at the date of the last follow-up or at the end of the observation period.

### Statistical analysis

The sample size (*n* = 38) was determined on the basis of the annual surgical volume of the medical center, with approximately 280 cancer-related GI surgeries per year. It was estimated that 50% of surgeries would not meet the inclusion criteria. Among the remaining patients (140 surgeries), 65% would not participate because of long travel distances or other reasons. Therefore, it was estimated that 13.5% of all surgeries performed each year were applicable for study participation. This estimate was derived retrospectively based on institutional data. The sample sizes were compared with those of similar studies [12]. Feasibility outcomes were summarized descriptively, with eligibility, recruitment, retention, and adherence rates reported as percentages and adverse events reported as counts. For QoL analysis, normality was assessed via the Shapiro–Wilk test, Q-Q plots, and histograms. Change scores accounted for baseline differences. Intergroup comparisons used one-tailed independent t-tests for normally distributed data, reflecting prespecified directional hypotheses that the prehabilitation intervention would result in superior outcomes compared with usual care, with no plausible expectation of clinically meaningful harm. To assess the robustness of these findings, additional two-tailed analyses were conducted as a sensitivity analysis. Welch’s test was applied for unequal variances, and Mann–Whitney U test was used as a nonparametric alternative. Whitin-group changes were analyzed with dependent two-tailed t-tests or Wilcoxon signed-rank tests when appropriate. Significance was set at *p* < 0.05, with *p* < 0.001 considered highly significant. Effect sizes were interpreted using Cohen’s *d* (small: 0.2; medium: 0.5; large: 0.8). Data are presented as means ± standard deviations (SD). Analyses were conducted via IBM SPSS Version 22.

Survival outcomes were measured in months. Kaplan—Meier estimates were used to visualize survival probabilities for the two groups, with 24-month survival probabilities extracted. Participants were censored at the last follow-up on June 15, 2025. No formal hypothesis testing (e.g., log-rank test) was performed. Survival analyses were conducted using R (version 4.4.1) with the survival and survminer R packages [13, 14]. Given the exploratory design and limited sample size, the results were interpreted descriptively without adjustment for multiple testing.

## Results

**Fig. 1:**
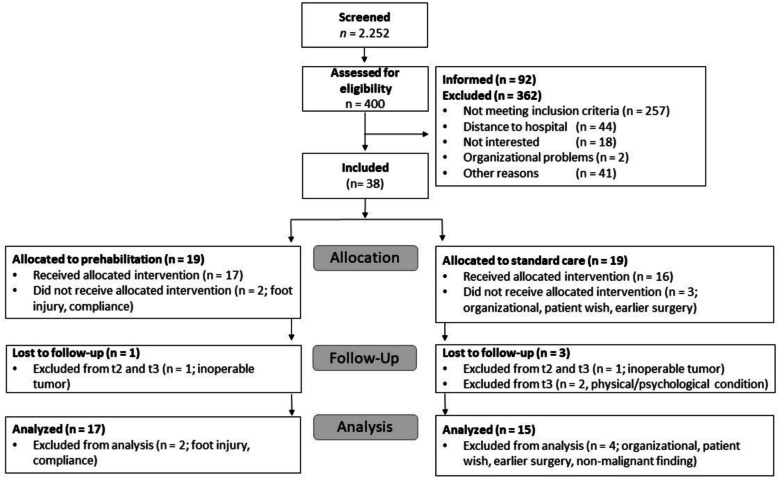
Patient flowchart

Between September 2022 and December 2023, a total of 2,252 patients were screened, of whom 400 were assessed for eligibility, 38 consented to participate and were assigned to either the prehabilitation group or the usual care group. Patient flow is displayed in Fig. 1. Six patients (prehabilitation: *n* = 2; usual care: *n* = 4) were excluded prior to analysis: One patient was found to have a non-malignant diagnosis at surgery and five did not complete the planned intervention. Reasons for non-completion were organizational issues, earlier surgery, foot injury, noncompliance and patient preference. Following group allocation, additional events affected data completeness: Two patients were deemed inoperable during surgery and were therefore excluded from follow-up assessments. Further, two participants missed a single measurement due to organizational reasons (e.g., earlier surgery or hospital discharge). These cases were retained in the analysis in accordance with the intention-to-treat principle, using all available data. In total, 32 patients were analyzed, with the final follow-up completed in April 2024. The patient cohort comprised a diverse range of diagnoses, with a large proportion of patients having CRC (58% of patients receiving prehabilitation; 73% of patients receiving usual care). A higher prevalence of advanced cancer stages was observed in the prehabilitation group (75% versus 40%). Relevant comorbidities were similarly distributed in both groups. Specific details will be listed elsewhere (Assenbaum L et al., not yet published). Some patients received neoadjuvant oncological treatment prior to inclusion in the study (42% prehabilitation, 60% usual care), albeit not within the designated intervention period. While nutritional risk [15] and concomitant medication were similar between groups, more patients in the prehabilitation group had handgrip strength values below their healthy average. The baseline characteristics of the study population are presented in Table 1.

**Table 1:** Baseline characteristics of the study population

	**Prehabilitation (*n* = 17)**	**Usual care (*n* = 15)**
Age^#^ [years]	63 (40–80)	59 (37–84)
Sex [n] (%) Male/Female	12 (75)/5 (25)	7 (53)/8 (47)
BMI^#^ [kg/m^2^]	26.7 (20–36)	26.4 (18–37)
Tumor localization [n] (%)		
Esophagus	2 (12)	3 (20)
Colon	4 (23)	7 (46)
Rectosigmoidal tract	1 (6)	1 (7)
Rectum	5 (29)	3 (20)
Pancreas	2 (12)	1 (7)
Small intestine	3 (18)	0
UICC [n] (%)		
Early stages (I—II)	5 (29)	9 (60)
Advanced stages (III – IV)	12 (71)	6 (40)
Initial diagnosis [n] (%) Yes/No	17 (100)/0	14 (93)/1 (7)
Previous therapy [n] (%)		
Surgery	2 (12)	2 (13)
Systemic therapy^a^	7 (41)	7 (47)
Chemoradiation	2 (12)	5 (33)
Irradiation primarius/metastases	2 (12)/1 (6)	1 (7)/0
None	10 (59)	7 (40)
Type of Surgery [n] (%) Laporoscopic/Open	9 (53)/8 (47)	12 (80)/3 (20)
Stoma [n] (%) Yes/No	4 (24)/13 (76)	5 (33)/10 (67)
Comorbidities [n] (%) Yes/No	14 (82)/3 (18)	11 (73)/4 (27)
Polypharmacy [n] (%) ≥ 5/< 5 medications	3 (18)/14 (82)	3 (20)/12 (80)
Risk for malnutrition [n] (%) NRS ≥ 3	3 (18)	3 (20)
Handgrip strength [n] (%) < healthy average (by age/sex/height)^b^	9 (53)	6 (40)
Smoking status [n] (%) No/Former/Current	8 (47)/7 (41)/2 (12)	7 (47)/2 (13)/6 (40)
Alcohol consumption [n] (%) No/Regular/Sometimes	6 (35)/2 (12)/9 (53)	3 (20)/4 (27)/8 (53)

*Abbreviations*: *BMI* body mass index (calculated as weight in kilograms divided by height in meters squared), *UICC* Union for International Cancer Control, *NRS* Nutritional Risk Screening

^#^median (range)

^a^chemotherapy or antibody therapy

^b^german reference population [16]; no significant differences between groups at baseline

### Feasibility and safety

The eligibility rate of all screened patients was 36%, the recruitment rate of the eligible patients was 27%, while the acceptance rate was 41% of all approached patients. The retention rates were 84% and 36% in relation to the eligible patients. The mean interval between t0 and t1 was 30 days (± 16.25), with 8.06 (± 3.6) of the 8.84 (± 4.73) planned exercise sessions being completed, and 4.76 (± 3.83) sessions completed at the intended training intensity. Thus, 95% of the patients adhered to the training program, and 59% of the sessions were conducted at the intended training intensity. Training sessions were skipped because of reduced general well-being (55%), patient wishes (22%), competing medical appointments (11%) and organizational reasons (11%). Patients with pancreatic and small intestine cancer attended fewer exercise sessions (mea*n* = 5 ± 0.63) than those with CRC (9.4 ± 3.26) or esophageal cancer (9 ± 4.0). Nutritional intervention was conducted in 16 patients with one session missing for organizational reasons, corresponding to 94% completed nutritional consultations. No serious adverse events occurred due to exercise. Two adverse events occurred during the intervention (one foot injury, one broken arm with surgery) but were not related to study participation.

### QoL

The results of the secondary outcome variables are depicted in Table 2. There was no significant group difference in the change scores in global QoL, PCS and MCS from baseline. The absolute values of PCS differed significantly between groups at t1 (one-tailed *p* = 0.04, *d* = 0.64), but this effect was not statistically significant in the two-tailed analysis. In both groups, the intragroup changes between t1, t2 and t3 were significant for QoL (t1 to t2/t2 to t3: Prehabilitation *p* < 0.05., *d* = 0.65/*d* = 0.81; Usual Care: *p* < 0.05, *d* = 0.91/*d* = 1.03) and PCS (t1 to t2/t2 to t3: Prehabilitation p < 0.001, *d* = 2.9/*d* = 1.6; Usual care: p < 0.001, d = 1.5/p = 0.003, *d* = 1.16). Additionally, the intragroup change for PCS was significant between t0 and t1 (*p* = 0.023, *d* = 0.61) and the intragroup change for MCS was significant between t1 and t2 (*p* = 0.003, *d* = 0.74) in the prehabilitation group.

**Table 2:** Secondary outcome variables

Outcome		Prehabilitation [*n* = 17]	Usual Care [*n* = 15]	Group difference	Δ change from baseline
		**Mean ± SD**	**Mean ± SD**	***p* value (1-tailed/2-tailed)**	**Mean group difference**
Global QoL^a^	t0	62.25 ± 15.62	57.77 ± 19.28	.24/.47	
	t1	66.65 ± 20.62	60.12 ± 16.40	.12/.25	3.19
	t2	42.71 ± 26.33*	34.03 ± 22.89*	.19/.37	1.91
	t3	61.98 ± 23.15*	61.35 ± 16.36*	.43/.87	- 3.30
PCS^b^	t0	44.60 ± 9.03	42.57 ± 9.80	.27/.54	
	t1	48.85 ± 6.58*	43.69 ± 9.67	.04*/.09	3.47
	t2	28.54 ± 7.32**	27.83 ± 6.52**	.40/.79	- .69
	t3	43.53 ± 9.27**	39.93 ± 9.28*	.16/.32	3.64
MCS^c^	t0	44.51 ± 10.96	46.55 ± 12.47	.31/.63	
	t1	46.34 ± 12.84	48.85 ± 10.75	.28/.57	- .98
	t2	46.66 ± 14.00*	44.47 ± 9.43	.32/.65	4.05
	t3	46.50 ± 12.10	50.04 ± 8.68	.20/.40	- .38

*Abbreviations*: *t0* surgical indication, *t1 *presurgery (± 1 day), *t2* hospital discharge (± 1 day); *t3*, 12 weeks post-discharge

^a^EORTC QLQ-C30

^b^physical health component summary score SF-36

^c^mental health component summary score SF-36

*p <.05, **p <.001; significant differences between groups (change scores) or significant intragroup change to previous measurement

### Survival

**Fig. 2:**
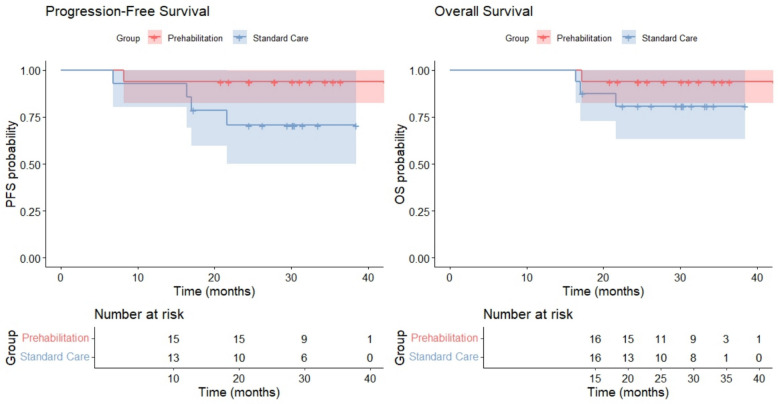
Progression free survival (PFS) and overall survival (OS). *N* = 4 deaths in total, *N* = 2 dropouts included in analysis

Among the 34 patients analyzed for survival, two had recurrent GI cancer (5.9%). These patients were included in the descriptive and survival analyses. A sensitivity analysis excluding recurrent cases revealed no meaningful differences in the overall results. The Kaplan–Meier estimate is displayed in Fig. 2 and based on 34 patients, including two dropouts of the usual care group. They were included in the analysis, as they missed study assessments for organizational reasons only. Overall, four patients died within two years. Owing to the low number of events and the high survival rate (more than one year for all), the estimate is associated with high uncertainty. At 24 months, the estimated OS was 93.7% [95% CI 0.83; 1.00] in the prehabilitation group and 77.9% [95% CI 0.58; 1.00] in the usual care group.

## Discussion

This investigation supports the feasibility and safety of exercise and nutritional prehabilitation in patients with GI cancer. The primary outcomes align with previous feasibility criteria [6], with an eligibility rate of 36% and a recruitment rate of 27%, meeting the predefined threshold of ≥ 20% suggested by Morielli et al. [17]. Recruitment challenges in this population are multifactorial, including the short interval between diagnosis and surgery, long travel distances to the oncological center, compromised physical status, and the psychological burden of a cancer diagnosis, all of which may contribute to selection bias. The type of diagnosis may further influence recruitment success. A systematic review similarly reported higher recruitment rates in CRC patients than in esophagogastric cancer patients, potentially reflecting differences in symptom burden [18]. No feasibility studies focusing on pancreatic cancer were identified in that review and while some other studies have examined intervention effects, feasibility outcomes remain underreported [19]. Adherence to overall exercise and nutritional intervention was high (95% and 94%, respectively), clearly exceeding commonly applied thresholds (≥ 60%–80%) [5]. However, adherence to the prescribed exercise intensity was lower (59%), likely due to disease- or treatment-related side effects. Patients with pancreatic and small intestine cancer attended fewer exercise sessions than those with CRC or esophageal cancer, likely due to tighter surgical schedules that reduced the available preoperative timeframe for exercise and due to increased symptom burden. However, evidence in pancreatic cancer remains scarce, with unclear exercise dosing and intensity, which are relevant factors for clinical applicability. In the absence of robust dose–response data for exercise interventions in oncological prehabilitation, studies on sarcopenia management in healthy older adults (≥ 60 years) may provide limited contextual reference. In these populations, moderate training intensities (51–69% of one-repetition maximum, 1-RM) were most effective for improving muscle morphology, whereas higher intensities (70–79% of 1-RM) predominantly improved muscle strength [20]. Furthermore, a recent meta-analysis revealed that a minimum weekly training volume of approximately 30 sets of full-body exercises (8–10 repetitions per set) is required to induce muscle hypertrophy [21]. However, applicability to patients with GI cancer is highly limited, as increased catabolic burden, treatment-related symptoms, and reduced exercise tolerance may substantially affect feasibility and training response. These parameters should therefore be interpreted with caution and not directly translated, but rather considered as a preliminary framework for future population-specific dose–response research. The absence of adverse events supports the safety of the intervention at the intended training intensity. Furthermore, the timeframe between surgical indication and operation appears applicable for patients with different GI cancer entities, providing a realistic period for intervention. However, the clinical integration of prehabilitation demands a high level of organizational coordination among the treating interdisciplinary teams. This point is underscored by the fact that the time to surgery was one important reason why some of the screened patients did not meet the inclusion criteria. A delay of surgery in favor of prehabilitation has gained increasing attention in recent studies [22, 23]. Other novel concepts, such as preoperative chemotherapy for locally advanced colon cancers [24] or neoadjuvant immune checkpoint inhibition with less impact on physical fitness, offer promising potential to enhance the realizability of training interventions [25, 26]. This may increase patient resilience, extend the timeframe available for prehabilitation and further improve the therapy response.

With respect to the patient-reported secondary outcomes, a modest preoperative improvement in PCS was observed in the prehabilitation group. While the between-group difference at t1 reached statistical significance using one-tailed testing, this effect was not confirmed under two-tailed testing. Nevertheless, a significant within-group increase in PCS was observed in the prehabilitation group between t0 and t1, suggesting a possible benefit of the program on perceived physical functioning prior to surgery. However, the observed modest benefit for physical health should be considered hypothesis-generating, requiring confirmation in an adequately powered randomized trial. No significant effects were observed for global QoL or mental well-being between groups, likely due to the short intervention period and persistent psychological stress before surgery. These results highlight the challenge of maintaining perceived physical health benefits in the perioperative period. Research has shown that longer-term interventions are more effective in improving QoL and psychological outcomes [27], but in surgical care, the timeframe is inherently constrained by operative scheduling. Importantly, the study was designed as a feasibility study and was not powered to detect clinically meaningful differences in patient-reported outcomes. The sample size was based on the practical capacity of the center rather than a formal power calculation for specific endpoints, and therefore all inferential comparisons should be interpreted as exploratory. Data on the effects of prehabilitation on cancer recurrence and OS in GI cancer patients remain limited [28, 29]. However, an investigation of CRC patients suggested improved 5-year disease-free survival for patients with stage III disease [30]. In the present study, exploratory survival analyses suggested a possible trend toward improved PFS and OS in the prehabilitation group; however, these findings must be interpreted with caution: The study was neither designed nor powered to detect survival differences, and the low number of events (four among 34 patients) introduces substantial statistical uncertainty and wide confidence intervals. Accordingly, Kaplan–Meier estimates, including 24-month OS rates, may convey greater precision than is warranted, particularly given the limited follow-up. Reporting median follow-up time and numbers at risk at relevant time points may provide more informative context for interpreting these results and for planning future adequately powered trials. Given the nonrandomized, patient-preference–based design, between-group comparisons are subject to potential selection bias related to unmeasured factors such as motivation and baseline physical fitness. In addition, there was a notable imbalance in disease stage, with a higher proportion of advanced-stage disease in the prehabilitation group (71% vs. 40%). This fact may have confounded outcomes, as disease stage is a known prognostic factor for both QoL and survival. This imbalance may reflect selection and referral biases, with patients who were more motivated or had a better performance status being more likely to enroll in the intensive prehabilitation program despite having more advanced disease. On the other hand, patients with advanced disease may have been preferentially referred to prehabilitation because of a perceived greater need for supportive care. Importantly, despite this imbalance, slightly more favorable patient-reported outcomes were observed in the prehabilitation group, suggesting that baseline differences alone are unlikely to fully explain the observed patterns. However, given the small sample size and nonrandomized design, all between-group effects must be interpreted as exploratory and hypothesis-generating. A key strength of this study is its comprehensive approach to investigate feasibility within a multimodal approach. The inclusion of high-risk patients and multiple disease entities enables a more inclusive assessment of feasibility, thereby improving clinical generalizability and reflecting routine clinical practice more accurately than highly selected cohorts do [6]. However, investigations in a single-center setting may not translate to centers without dedicated oncology-exercise facilities. While the study is adequate for assessing feasibility outcomes, the small sample size and the absence of randomization with potential selection bias clearly limit the interpretation of effects on patient-reported outcomes and survival. However, the decision to conduct a randomized controlled trial requires careful ethical consideration, given the clear potential benefits of exercise and nutritional prehabilitation [31].

As this investigation was designed to serve as a foundation for a larger trial, the next step will be to address patient-related barriers to participation and to develop tailored strategies that accommodate the diverse needs of individual patients. This may include flexible delivery models potentially through digital health or homebased support to reduce logistical barriers. Individualized monitoring strategies may be necessary to optimize training intensities according to the training target. Furthermore, a measurement of everyday activities prior to diagnosis would provide a better interpretation of training effects. As shown in latest results, prehabilitation should be combined with long-term postoperative exercise to achieve sustained benefits in both physical and mental health as well as PFS [32]. Such adaptations may improve recruitment and intervention fidelity, thereby enhancing the ability to capture the full potential benefits in future studies.

## Conclusion

Exercise and nutritional prehabilitation for GI cancer patients is feasible, safe and well-tolerated, even in a clinically diverse and high-risk population reflecting the real-world setting. Although underpowered to confirm survival or patient-reported benefits, the results support the conduct of a randomized trial in a multicenter setting to allow for a larger sample size that is adequately powered for clearly defined primary outcomes. Such a study should also address recruitment barriers and consider tailoring interventions to maximize short- and long-term outcomes.

## Data Availability

This article only includes summarized data of this study. The datasets analyzed during the current study are available from the corresponding author upon reasonable request.

## Abbreviations

BMI: Body mass index (calculated as weight in kilograms divided by height in meters squared)
CRC: Colorectal cancer
GI: Gastrointestinal
MCS: Mental health component score
NRS: Nutritional risk screening
OS: Overall survival
PCS: Physical health component score
PFS: Progression free survival
1-RM: One-repetition maximum
SD: Standard deviation
UICC: Union for International Cancer Control
QoL: Quality of Life
